# An *In Vitro* Co-culture Mouse Model Demonstrates Efficient Vaccine-Mediated Control of *Francisella tularensis* SCHU S4 and Identifies Nitric Oxide as a Predictor of Efficacy

**DOI:** 10.3389/fcimb.2016.00152

**Published:** 2016-11-25

**Authors:** Igor Golovliov, Helena Lindgren, Kjell Eneslätt, Wayne Conlan, Amandine Mosnier, Thomas Henry, Anders Sjöstedt

**Affiliations:** ^1^Laboratory for Molecular Infection Medicine Sweden, Department of Clinical Microbiology, Clinical Bacteriology, and Umeå UniversityUmeå, Sweden; ^2^National Research Council Canada, Institute for Biological SciencesOttawa, ON, Canada; ^3^Centre International de Recherche en Infectiologie, Institut national de la santé et de la recherche médicale, U1111Lyon, France

**Keywords:** *F. tularensis* SCHU S4, *in vitro* co-culture model, mouse immune response, correlates of protection

## Abstract

*Francisella tularensis* is a highly virulent intracellular bacterium and cell-mediated immunity is critical for protection, but mechanisms of protection against highly virulent variants, such as the prototypic strain *F. tularensis* strain SCHU S4, are poorly understood. To this end, we established a co-culture system, based on splenocytes from naïve, or immunized mice and *in vitro* infected bone marrow-derived macrophages that allowed assessment of mechanisms controlling infection with *F. tularensis*. We utilized the system to understand why the *clpB* gene deletion mutant, Δ*clp*B, of SCHU S4 shows superior efficacy as a vaccine in the mouse model as compared to the existing human vaccine, the live vaccine strain (LVS). Compared to naïve splenocytes, Δ*clpB*-, or LVS-immune splenocytes conferred very significant control of a SCHU S4 infection and the Δ*clpB*-immune splenocytes were superior to the LVS-immune splenocytes. Cultures with the Δ*clpB*-immune splenocytes also contained higher levels of IFN-γ, IL-17, and GM-CSF and nitric oxide, and T cells expressing combinations of IFN-γ, TNF-α, and IL-17, than did cultures with LVS-immune splenocytes. There was strong inverse correlation between bacterial replication and levels of nitrite, an end product of nitric oxide, and essentially no control was observed when BMDM from iNOS^−/−^ mice were infected. Collectively, the co-culture model identified a critical role of nitric oxide for protection against a highly virulent strain of *F. tularensis*.

## Introduction

*Francisella tularensis* is a highly virulent facultative intracellular bacterium causing the severe disease tularemia in many mammalian species (Sjöstedt, 2007). Two subspecies are common human pathogens, subspecies *tularensis* (type A), which causes disease with high mortality if untreated, and the less aggressive subspecies *holarctica* (type B), which despite its lower virulence may cause serious illness in humans. Tularemia is widespread over the Northern hemisphere and a significant health problem, in particular in certain parts of Scandinavia, in parts of Eastern Europe, and in Turkey, but a rather uncommon disease in most other parts of the world. The live vaccine strain (LVS) is a human vaccine strain, which confers efficacious protection against laboratory-acquired infection, as demonstrated by the fact that the incidence of tularemia among the laboratory staff decreased by 95% after its introduction (Burke, 1977). Despite this success, studies on volunteers in the 1960s revealed that it did not confer efficacious protection against aerosol infection [reviewed by Conlan et al. (Conlan, 2011)]. Therefore, there is a need for development of more efficacious *Francisella* vaccines and previously, we analyzed if defined mutants of SCHU S4 (type A) could serve as such vaccine candidates and identified that the Δ*clpB* mutant conferred superior efficacy compared to LVS, despite that the former was more attenuated (Kadzhaev et al., 2009; Conlan et al., 2010; Ryden et al., 2012). It encodes an AAA+ chaperone, although the exact function in *F. tularensis* is still unknown.

Due to the essential role of CMI for host protection against tularemia, a thorough understanding of its characteristics will be necessary to identify how the infection is controlled. There is accumulating evidence that the protective mechanisms are effectuated via a complex interplay of multiple T cell subsets and other immune mechanisms, and not a single specific immune activity (Elkins et al., 2007; De Pascalis et al., 2008, 2012, 2014; Cowley and Elkins, 2011; Eneslätt et al., 2011, 2012; Mahawar et al., 2013; Griffin et al., 2015). Thus, the mechanisms cannot be delineated using simple proliferation assays but will require assays that closely mimic the *in vivo* situation. Therefore, new models are needed to better understand protective mechanisms and validate potential correlates of protective immunity. In this regard, a functional *in vitro* splenocyte-bone marrow-derived macrophage (BMDM) co-culture assay for measuring potential correlates of protection for tularemia vaccines was developed and substantial work has been performed with the aim to establish correlates of protection against *F. tularensis* (Cowley and Elkins, 2003; Cowley et al., 2005; Collazo et al., 2009; Elkins et al., 2011; De Pascalis et al., 2012, 2014; Mahawar et al., 2013; Griffin et al., 2015). Pascalis et al. showed that the greater efficacy of LVS vs. attenuated derivatives correlated with relative bactericidal activity in the co-culture system (De Pascalis et al., 2012). The relevance of such co-culture assays has to some extent been validated by the demonstration that the identified immune molecules play important roles *in vivo* (Kurtz et al., 2013; Melillo et al., 2013, 2014). A limitation of the published work using the splenocyte-BMDM co-culture model has been the extensive use of the attenuated LVS strain and there are very few studies, which have utilized fully virulent *F. tularensis* (Mahawar et al., 2013; Griffin et al., 2015).

The absence of any demonstrable correlate(s) of protection is an obstacle to the licensure of *F. tularensis* vaccines. Tularemia is in most countries an unusual disease and even in endemic areas, it appears with very irregular intervals (Sjöstedt, 2007). Therefore, efficacy in a vaccinated population will not be possible to evaluate. Moreover, although challenge studies of volunteers were performed in the 1950s (Saslaw et al., 1961a,b), such studies, in view of the severity of respiratory tularemia, are very unlikely to be approved today. Therefore, a tularemia vaccine, as well as other vaccines against unusual, aggressive diseases, need to be evaluated according to the FDA Animal Rule (Snoy, 2010). The regulation stipulates that efficacy testing can be performed exclusively using animal models, provided that the mechanisms of action of the vaccine are sufficiently well characterized to permit extrapolation of efficacy to humans. Only one vaccine has so far been approved according to the Animal Rule (US. Food Drug Administration., 2015).

Efficacy of experimental tularemia vaccines has been demonstrated using mouse, rat, rabbit, and non-human primate models, but none of these have been approved by FDA. Thus, models to test tularemia vaccine efficacy will require further characterization before they can be approved, e.g., the identification of correlates of immunity and protection. This would also include models that would not necessitate lethal animal challenges. Therefore, we wanted to investigate if a co-culture assay based on infection of mouse bone marrow-derived macrophages could be utilized to assess vaccine efficacy against the highly virulent SCHU S4 strain. In view of the previously described superior vaccine-mediated protection conferred by the vaccine candidate Δ*clp*B compared to LVS (Kadzhaev et al., 2009; Conlan et al., 2010; Ryden et al., 2012), we asked if this superior trait also was reflected using the co-culture assay. Indeed, we observed that Δ*clpB*-immune splenocytes conferred superior control of the SCHU S4 strain compared to LVS-immune splenocytes and efficacy strongly correlated with levels of nitric oxide.

## Materials and methods

### Bacterial strains

*F. tularensis* LVS was originally obtained from the American Type Culture Collection (ATCC 29684). *F. tularensis* strain SCHU S4 (*F. tularensis* subsp. *tularensis*) was obtained from the *Francisella* Strain Collection of the Swedish Defense Research Agency, Umeå, Sweden. The generation in our laboratory of the Δ*clpB* strain by allelic replacement of the *clp*B gene, a procedure that did not introduce any recombinant DNA in the strain, has been described previously (Conlan et al., 2010). All bacteriological work related to the SCHU S4 strain was carried out in a biosafety level 3 facility certified by the Swedish Work Environment Authority.

### Animals

In the experiments, Balb/c or C57/BL6 mice obtained from Charles River, Germany were used. When required, mice were immunized with a dose of approximately 5 × 10^3^ CFU of the LVS or the Δ*clpB* strain subcutaneously. The ensuing infection resulted in no or very mild objective symptoms between days 4 and 6 of the infection. Ethical approval for all of the described mouse experiments was obtained from the Ethical Committee on Animal Research, Umeå, Sweden, A99-11, and A67-14 and the University of Lyon, France (CECCAPP) under the protocol number #ENS_2012_061.

### Generation of BMDM

Bone marrow was flushed from femurs of Balb/c or C57BL6/J mice with Dulbecco's Modified Eagle Medium (DMEM). Typically, femurs from two mice were used in each experiment. Cells were washed, a single-cell suspension was prepared by gentle pipetting in complete DMEM [DMEM supplemented with 10% of heat-inactivated fetal bovine serum, 0.2 of mM L-glutamine, (Life Technologies), 1 mM of HEPES buffer (Life Technologies), and 50 μM of β-mercaptoethanol), and 10% of L-929 conditioned medium. After counting, 5 × 10^6^ BMDM in 10 ml of DMEM were added to a 10 cm Petri dish and incubated at 37°C and 5% CO_2_. After 3 days, 5 ml of complete DMEM containing L-929 conditioned medium were added. After 7 days of total incubation, medium was removed, 10 ml of cold PBS/10 mM EDTA was added and the dishes were incubated on ice for 30 min. The macrophages were carefully collected by pipetting, centrifuged, resuspended in complete DMEM and 5 × 10^5^ cells per well were added to 24-well plates and incubated overnight at 37°C and 5% CO_2_ and then used in the co-culture assay. The number of viable BMDM was determined after trypan blue staining using Vi-CELL XR cell viability analyzer (Beckman Coulter).

### Splenocyte preparation

Four to 5 weeks following immunization of Balb/c or C57/BL6 mice, spleens were aseptically removed from mice and cells were released by gently squeezing the organs with an L-shaped needle. Splenocytes from three mice were used for each group. Splenocytes were prepared essentially as described (Bosio and Elkins, 2001). A single-cell suspension was prepared, centrifuged and erythrocytes were lysed with ammonium chloride. Cells were washed by centrifugation with PBS + 2% FBS, and resuspended finally in complete DMEM. The number of viable splenocytes was determined after trypan blue staining using Vi-CELL XR cell viability analyzer (Beckman Coulter). The splenocytes were then used either in the co-culture assay or in the *in vitro* recall response assay.

### Infection of the BMDM in the co-culture assay

Bacteria were grown overnight on Gc-agar plates, resuspended in PBS, and added to the BMDM monolayer at an MOI of 1:5 (bacteria:BMDM). After uptake for 2 h, medium was removed and the macrophage monolayer was washed twice using sterile PBS at RT. One ml of cDMEM containing 20 μg/ml gentamicin was added to each well, plates were incubated for an additional 45 min and washed twice with PBS. Following the last wash, each BMDM monolayer was overlaid with 1 ml of complete DMEM with 2.5 × 10^6^ of congenic splenocytes, giving a ratio BMDM:splenocytes of 1:5. Bacterial counts were determined by lysis of the cells and plating of serial dilutions (Golovliov et al., 2003). In indicated cultures, 1 mM of N^G^-monomethyl-L-arginine (NMMLA) was added simultaneously with the splenocytes and maintained for the remainder of the experiment.

### Optimization of the *in vitro* splenocyte-BMDM co-culture model

The *in vitro* co-culture assay was previously established for the LVS strain (Bosio and Elkins, 2001; Cowley and Elkins, 2003). We now optimized the assay so it reproducibly could be used for the SCHU S4 strain as well. This involved the optimization of the ratio between T-cells with the antigen-presenting cells. It was found that a minimum of 4 × 10^5^ BMDM per well and 5-fold more immune splenocytes, i.e., a ratio of 1:5, resulted in the best control of both intracellular LVS and SCHU S4. When the ratio was 1:1, the growth inhibition of LVS and SCHU S4 was on average 0.7 ± 0.1 and 1.3 ± 0.4 log_10_ CFU, respectively, less than the growth inhibition using the ratio of 1:5. A ratio of 1:10 led to results similar to those using the 1:5 ratio. Also, the role of the bacterial MOI was investigated and bacterial uptake was determined by lysis of the cultures after addition of bacteria for 120 min and it was determined that an MOI of 1:5 (bacteria/BMDM) resulted in optimal growth inhibition.

### *In vitro* recall response and lymphocyte proliferation assay

Splenocytes were prepared from naïve mice or from mice immunized with LVS or Δ*clp*B as described above and seeded at 2 × 10^5^ in 200 μL of complete DMEM supplemented with 50 μM of β-mercaptoethanol per well in 96-well plates. Splenocytes were stimulated with a 1:1 mix of formalin-fixed LVS (ffLVS) and formalin-fixed SCHU S4 (ffSCHUS4) antigen at a final concentration of 0.5 bacteria/splenocyte or without antigen and incubated in a humidified atmosphere with 5% CO_2_ at 37°C. After 3 days the proliferative response of the splenocytes was detected by measuring the [^3^H]-thymidine incorporation as previously described (Ericsson et al., 1994) or analyzed by FACS as described below.

### Nitrite measurement

The amount of ${\text{NO}}_{2}^{-}$ in the culture supernatants after 72 h incubation was determined by use of the Griess reagent (Giovannoni et al., 1997). One hundred microliter of the culture supernatant was mixed with 100 μl each of the Griess reagents, p-Aminobenzenesulfonamide (58 mM in 5% H_3_PO_4_), and 2,6,8-Trihydroxypurine (3.9 mM) (Sigma). After 10 min of incubation at RT, the absorbance at 540 nm was recorded. The concentration of ${\text{NO}}_{2}^{-}$ was determined by preparing a standard curve of sodium nitrite. The lower limit of detection in the assay was 1.0 μM, i.e., below that concentration, medium spiked with nitrite showed an absorbance indistinguishable from that of buffer alone.

### Multiplex cytokine analysis

Cell culture supernatants, 50 μL/well, were collected from the same cell cultures as used for assessment of intracellular bacterial replication and stored frozen at −80°C until analyzed using a commercial 23-plex kit (catalog #M60009RDPD) or a custom-made 9-plex kit according to the manufacturer's instructions with a Bio-Plex 200 system (BioRad Laboratories Inc, Hercules, CA, USA). The 9-plex kit contained following cytokines: IL-2, IL-6, IL-12p40, IL-17, IFN-γ, MIP-1β, GM-CSF, RANTES, and TNF-α.

### Flow cytometry analysis of surface markers and intracellular cytokine staining

Cells were collected after 72 h of incubation from either the lymphocyte proliferation assay or from the co-culture assay. Non-adherent cells were transferred to a new plate and 5 μg/mL of Brefeldin A was added. Four h later, plates were centrifuged for 3 min at 500 × g and supernatants were removed. After blocking of the Fc γ receptors with CD16/CD32 mouse BD Fc block (clone 2.4G2, BD Biosciences), cells were labeled with cell surface marker monoclonal antibodies (mAb) and conjugated intracellular cytokine mAb as recommended by BD Biosciences. The following mAb conjugates were used: CD3-APCCy7 (clone 17A2, BD Biosciences), CD4-AF700 (clone RM4-5, BD Biosciences), CD8-PerCPCy5.5 (clone 53-6.7, BD Biosciences), IFNγ-PE-CF594 (clone XMG1.2, BD Biosciences), TNF-α-Brilliant violet 421 (clone MP6-XT22, BioLegend), IL17A-PE/Cy7 (clone TC11-18H10.1, BD Biosciences). Aqua Viability Dye (Molecular Probes/Invitrogen) was added to distinguish live and dead cells. Cells were acquired using an LSRII flow cytometer (BD Biosciences) with FACSDiva software (BD Biosciences). Results were analyzed using FlowJo software (Tree Star).

### Data analysis and statistical methods

Two sample 2-tailed *t*-test, Mann-Whitney U or for paired data, Wilcoxon's signed rank test, were used to identify significant differences (*P* < 0.05) between data sets. To analyze correlation between data sets, Spearman's rank correlation test was used. A correlation with a coefficient (RS) above 0.4 was considered to demonstrate significant correlation, and a coefficient above 0.7 was considered to indicate strong correlation. When multiple experiments formed the basis for the analysis, the cumulative data were presented as boxplots. In each boxplot, the line through each box shows the median, with quartile one, and three as the lower and upper limits of each box. The end of the vertical lines indicates maximum and minimum values, respectively.

## Results

### Splenocyte recall response

**Figure 1:**
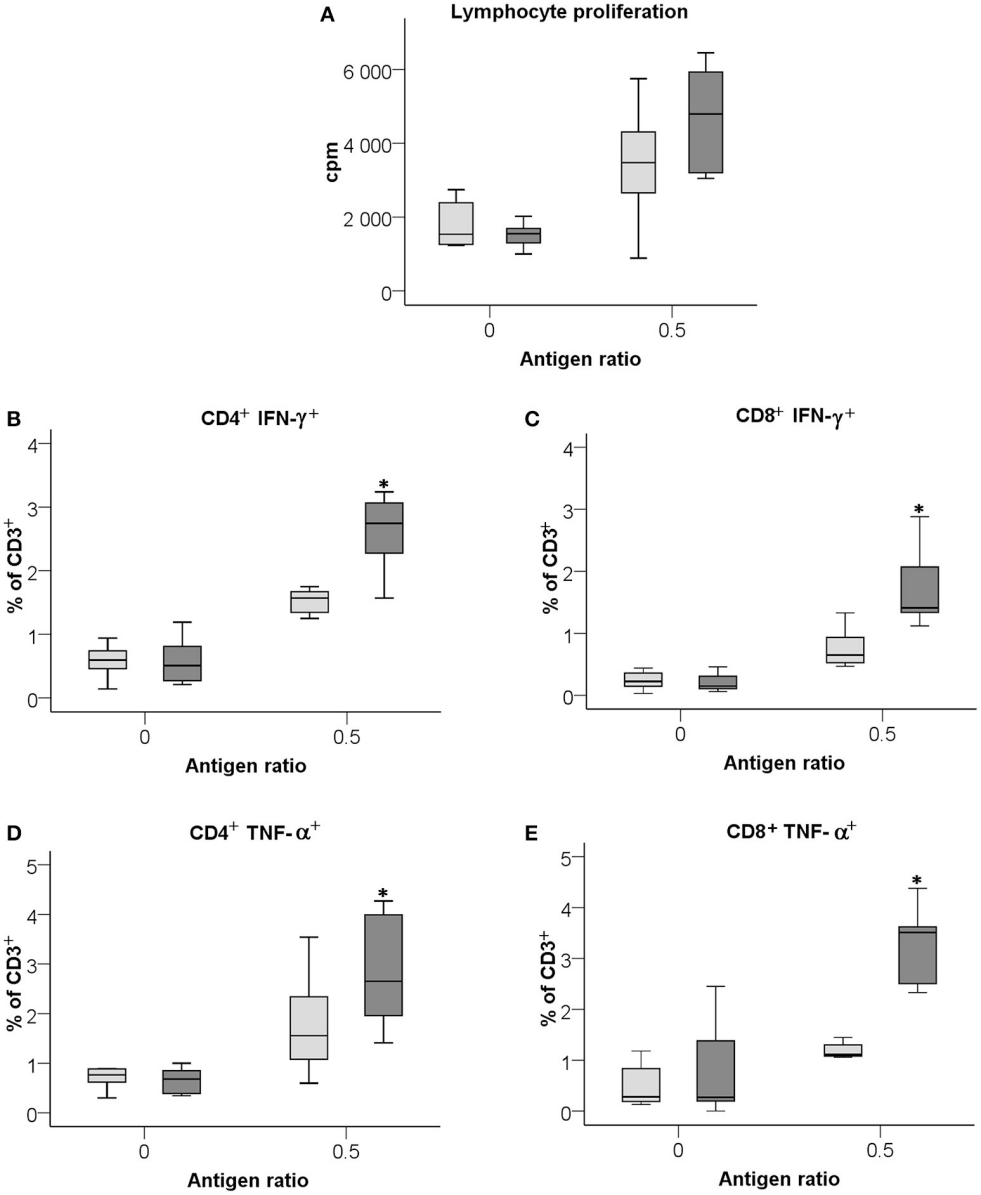
**Recall responses of LVS- (light gray boxes) or Δ***clp***B-immune (dark gray boxes) splenocytes analyzed with regard to lymphocyte proliferative responses (A),** CD4^+^ T cells expressing IFN-γ **(B)**, CD8^+^ T-cell expressing IFN-γ **(C)**, CD4^+^ T cells expressing TNF-α **(D)**, and CD8^+^ T-cell expressing TNF-α **(E)** Antigen ratio is expressed as number of killed bacteria/splenocyte. Results shown represent data from 3 experiments. Levels in cultures with naïve splenocytes were consistently <1500 cpm and showed no differences with or without antigen. Asterisks indicate *P* < 0.05 according to Wilcoxon's signed rank test for the pair-wise comparisons between LVS- and Δ*clp*B-immune data for each antigen ratio.

Splenocytes from mice immunized with LVS or Δ*clp*B were stimulated with formalin-killed *F. tularensis* antigen *in vitro* to measure the recall response of the T-cells with regard to proliferation and expression of cytokines. To accommodate for the different backgrounds of the two strains, a mixture prepared of equal amounts from LVS and SCHU S4 was analyzed to assess the immune reactivity. Both LVS- and Δ*clp*B-immune Balb/c splenocytes responded with very prominent proliferative responses to the antigen and the responses were stronger, although not significantly stronger, of Δ*clp*B- compared to LVS-immune splenocytes (*P* = 0.16; Figure 1A). Intracellular staining for IFN-γ and TNF-α showed that the percentages of CD4^+^ and CD8^+^ T-cells expressing the respective cytokine were significantly higher (*P* < 0.05) among Δ*clp*B- than LVS-immune splenocytes (Figures 1B–E).

Overall, the results show that Δ*clp*B-immune splenocytes demonstrated more prominent recall responses *in vitro* to the *F. tularensis* antigen than did LVS-immune splenocytes.

### Growth inhibition of *F. tularensis* LVS and cytokine production conferred by LVS- or Δ*clp*B-immune splenocytes

**Figure 2:**
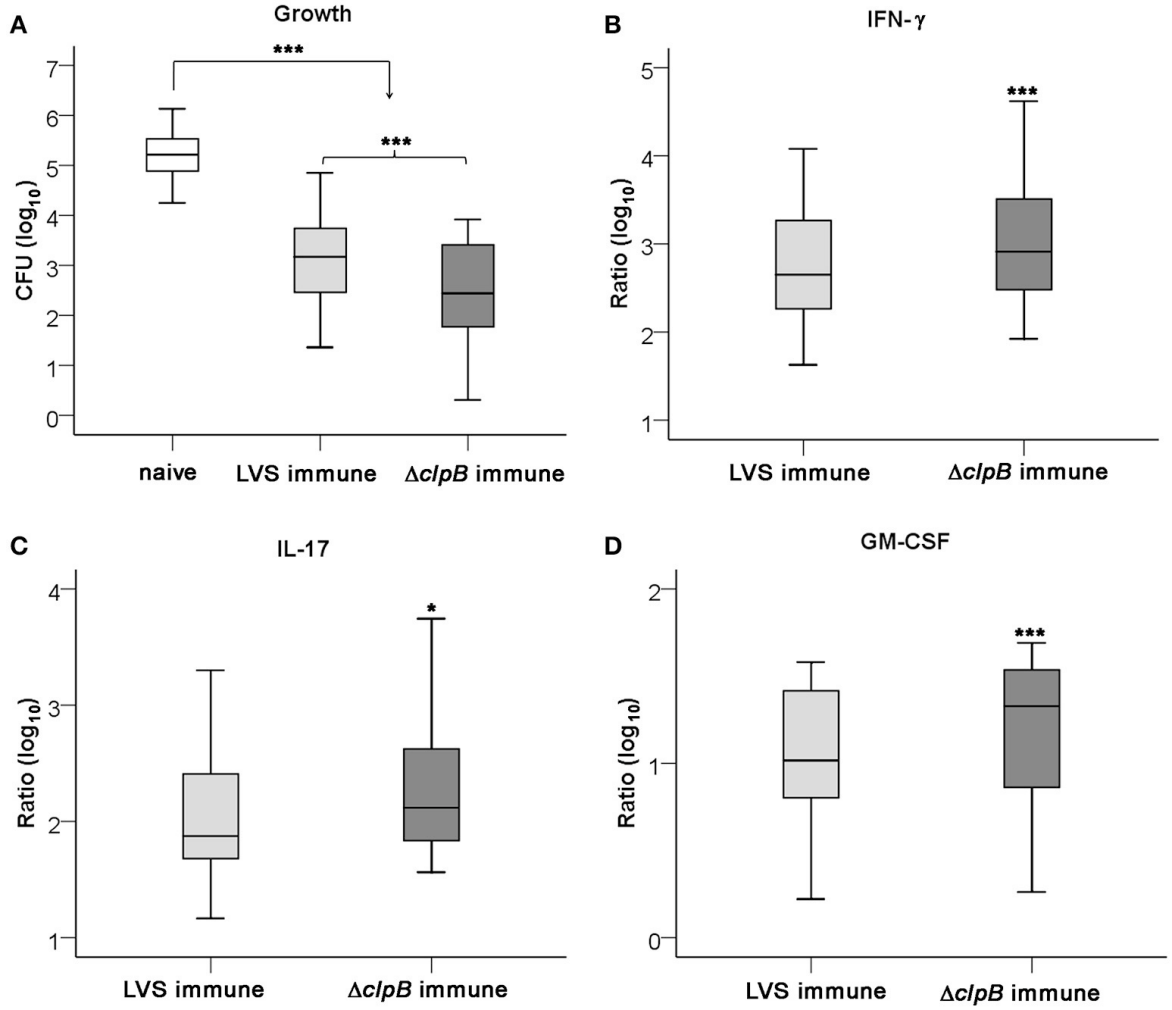
**Growth of ***F. tularensis*** LVS and cytokine accumulation in the co-culture assay**. Growth of LVS in cultures with naïve (white boxes), LVS-immune (light gray boxes) or Δ*clp*B-immune (dark gray boxes) splenocytes was determined after 72 h **(A)** and the accumulated levels of IFN-γ **(B)**, IL-17 **(C)**, and GM-CSF **(D)** in these cultures. Results shown represent data from 16 experiments in graph A and 11 experiments in graphs **(B–D)**. Growth was calculated as the log_10_ CFU of cultures at 72 h subtracted with the log_10_ CFU of cultures at 0 h [median and IQ one and three 2.3 (2.1, 3.2)]. In graphs **(B–D)**, the values are expressed as log_10_ fold-regulation of the indicated cytokine in relation to the concentration in cultures with naïve splenocytes. ^*^P < 0.05, ^***^P < 0.001 according to Wilcoxon's signed rank test. In graphs **(B–D)**, the significances indicate the pair-wise comparisons between LVS- and Δ*clp*B-immune data for each cytokine and in graph **(A)** for the indicated pair-wise comparisons.

The capacity of splenocytes from LVS- or Δ*clp*B-immunized mice to inhibit the intracellular growth of LVS in splenocyte-BMDM co-cultures was assessed. Addition of either type of immune splenocyte to the cultures significantly inhibited growth of LVS better than did naïve splenocytes, the median growth and the interquartile (IQ) one and three for all experiments performed were 3.2 [2.4, 3.8] and 2.4 [1.7, 3.4] log_10_ CFU for LVS-immune splenocytes and Δ*clp*B-immune splenocytes, respectively, vs. 5.2 [4.9, 5.6] log_10_ CFU for naïve splenocytes (*P* < 0.001 for immune *vs*. naïve). The Δ*clp*B-immune splenocytes showed superior control compared to LVS-immune splenocytes, *P* < 0.001 (Figure 2A). Not only growth inhibition was more prominent in cultures with Δ*clp*B-immune than LVS-immune splenocytes, but also levels of IFN-γ, IL-17, and GM-CSF in the culture supernatants were higher (*P* < 0.001; *P* < 0.05 and *P* < 0.001, respectively; Figures 2B–D).

In total, levels of 23 cytokines were analyzed in the supernatants from the co-cultures and 17 of those were found to be upregulated more than 2-fold in cultures with either of the immune splenocytes vs. cultures with naïve splenocytes, whereas MIP-1α was the sole cytokine being suppressed in cultures with immune splenocytes (Table S1). It was further analyzed how the cytokine levels of the supernatants correlated to the degree of protection, i.e., growth inhibition. Also, the correlations between the levels of the 23 cytokines and IFN-γ were analyzed. Growth inhibition was highly correlated (*P* < 0.01) to IFN-γ, GM-CSF, IL-10, IL-13, MIP-1α, and RANTES and among these cytokines, GM-CSF, MIP-1α, and RANTES were highly correlated (*P* < 0.01) to IFN-γ (Table 1). In addition, IFN-γ was highly correlated (*P* < 0.01) to IL-17, IL-6, IL-12p40, eotaxin, G-CSF, MCP-1, and MIP-1β (Table 1). From the 23 cytokines, 9 cytokines were chosen for use in subsequent analyses, since they all discriminated between levels in cultures with LVS- vs. Δ*clp*B-immunized splenocytes.

**Table 1:** **Correlations between growth inhibition, nitrite levels, IFN-γ levels and levels of other cytokines**.

	**Growth inhibition^a^**	**IFN-γ^b^**	**Nitrite^c^**
Growth inhibition	1.000	0.481^**^	0.679^**^
IFN-γ	0.481^d^^**^	1.000	0.525^**^
Nitrite	0.679^**^	0.525^**^	1.000
GM-CSF	0.356^**^	0.447^**^	0.435^**^
IL-10	0.499^**^	−0.007	0.560^**^
MIP-1α	0.576^**^	0.727^**^	0.238
IL-13	0.672^**^	0.566^**^	0.440^*^
RANTES	0.380^**^	0.718^**^	0.643^**^
IL-17	0.145	0.697^**^	0.241
IL-6	0.281^*^	0.898^**^	0.416^**^
IL-12(p40)	0.252	0.607^**^	0.488^**^
Eotaxin	0.353^*^	0.441^**^	0.186
G-CSF	0.066	0.664^**^	−0.244
MCP-1	0.228	0.713^**^	−0.450^*^
MIP-1β	0.307^*^	0.495^**^	0.342^*^

a Growth inhibition of F. tularensis LVS in cultures with LVS-immune or ΔclpB-immune splenocytes vs. naïve splenocytes in the in vitro assay after 72 h.

b Levels of cytokines measured in the supernatants of the in vitro assay after 72 h.

c Levels of nitrite measured in the supernatants of the in vitro assay after 72 h.

d Spearman's rank correlation coefficient between indicated combination.

* P < 0.05;

** P < 0.01.

In summary, several of the cytokines, including IFN-γ, strongly correlated to growth inhibition. Superior containment of LVS bacteria was observed in cultures with Δ*clp*B- vs. LVS-immune cells. The cytokine profile in the cultures with immune splenocytes reflected a Th1-type immune response.

### Characterization of immune splenocytes from the LVS-infected co-cultures

**Figure 3:**
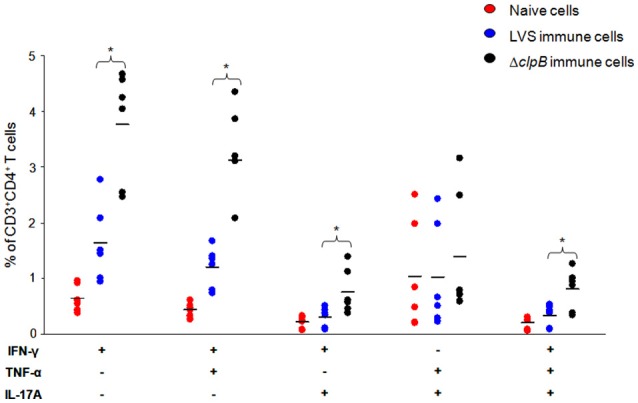
**Percentage of CD4^**+**^ and CD8^**+**^ T-cells expressing IFN-γ, IFN-γ, and TNF-α, or TNF-α and IL-17, or all three cytokines in cultures with naïve (red circles), LVS-immune (blue circles) or Δ*clp*B-immune (black circles) splenocytes incubated with LVS-infected BMDM for 72 h**. The percentages are expressed in relation to the CD3^+^ population. Results shown represent data from three experiments. ^*^ *P* < 0.05 according to Wilcoxon's signed rank test for the pair-wise comparisons between LVS- and Δ*clp*B-immune data for each cytokine combination.

To better understand the enhanced ability of the Δ*clp*B-immune splenocytes to control the replication of *F. tularensis* LVS, the percentage of IFN-γ-, TNF-α-, and IL-17-producing T cells were quantified by FACS analysis. IL-17 has in several publications been found to correlate to protection against *F. tularensis* (Lin et al., 2009; Mahawar et al., 2013). Analysis was performed on non-adherent cells from the cultures after 72 h of incubation. There were higher percentages of CD4^+^ and CD8^+^ T cells expressing IFN-γ in cultures from the immunized groups *vs*. the naïve group (*P* < 0.05) and among the immunized groups, cultures with Δ*clp*B-immune splenocytes expressed higher percentages (*P* < 0.05) of IFN-γ-positive cells among both the CD4^+^ and CD8^+^ subsets (Figure 3). Levels of cells expressing either IL-17 or TNF-α were similar between the immunized groups and the naïve group. Some studies have demonstrated that polyfunctional T cells demonstrate good correlation with host protection (Darrah et al., 2007; Derrick et al., 2011) and we identified such *F. tularensis*-specific T cells as part of the human immune response (Eneslätt et al., 2012). Also in the splenocyte-BMDM co-culture model, we observed the presence of polyfunctional *F. tularensis*-specific T cells, since in cultures with immune splenocytes, CD4^+^ and CD8^+^ T cells expressing IFN-γ and IL-17 or IFN-γ and TNF-α were significantly increased compared to cultures with naïve cells (*P* < 0.05). In all instances, the frequencies of the bifunctional T cells were significantly higher in cultures with Δ*clp*B-immune compared to LVS-immune splenocytes (*P* < 0.05) (Figure 3). Also, trifunctional CD4^+^ and CD8^+^ T cells were increased in cultures with immune splenocytes (*P* < 0.05) and, again, more frequent among Δ*clp*B-immune compared to LVS-immune splenocytes (*P* < 0.05; Figure 3). Median fluorescence intensity (MFI) of the immune splenocytes was analyzed for IFN-γ, IL-17, and TNF-α. Whereas, there were no significant differences for the latter two cytokines, Δ*clp*B-immune splenocytes demonstrated significantly higher MFI of IFN-γ expression among the CD4^+^ T cell populations than did LVS-immune splenocytes, median levels were 2430 [1890, 7710] vs. 1210 [670, 3930] (*P* < 0.05).

Collectively, the data demonstrated that there were qualitative and quantitative differences between Δ*clp*B- and LVS-immune splenocytes with regard to cytokine expression, since the Δ*clp*B-immune population contained higher frequencies of polyfunctional CD4^+^ and CD8^+^ T cells expressing IFN-γ in combination with IL-17 and/or TNF-α and also higher MFI of the IFN-γ-expressing CD4^+^ T cells.

### Growth inhibition of *F. tularensis* SCHU S4 and cytokine production conferred by LVS- or Δ*clp*B-immune splenocytes

**Figure 4:**
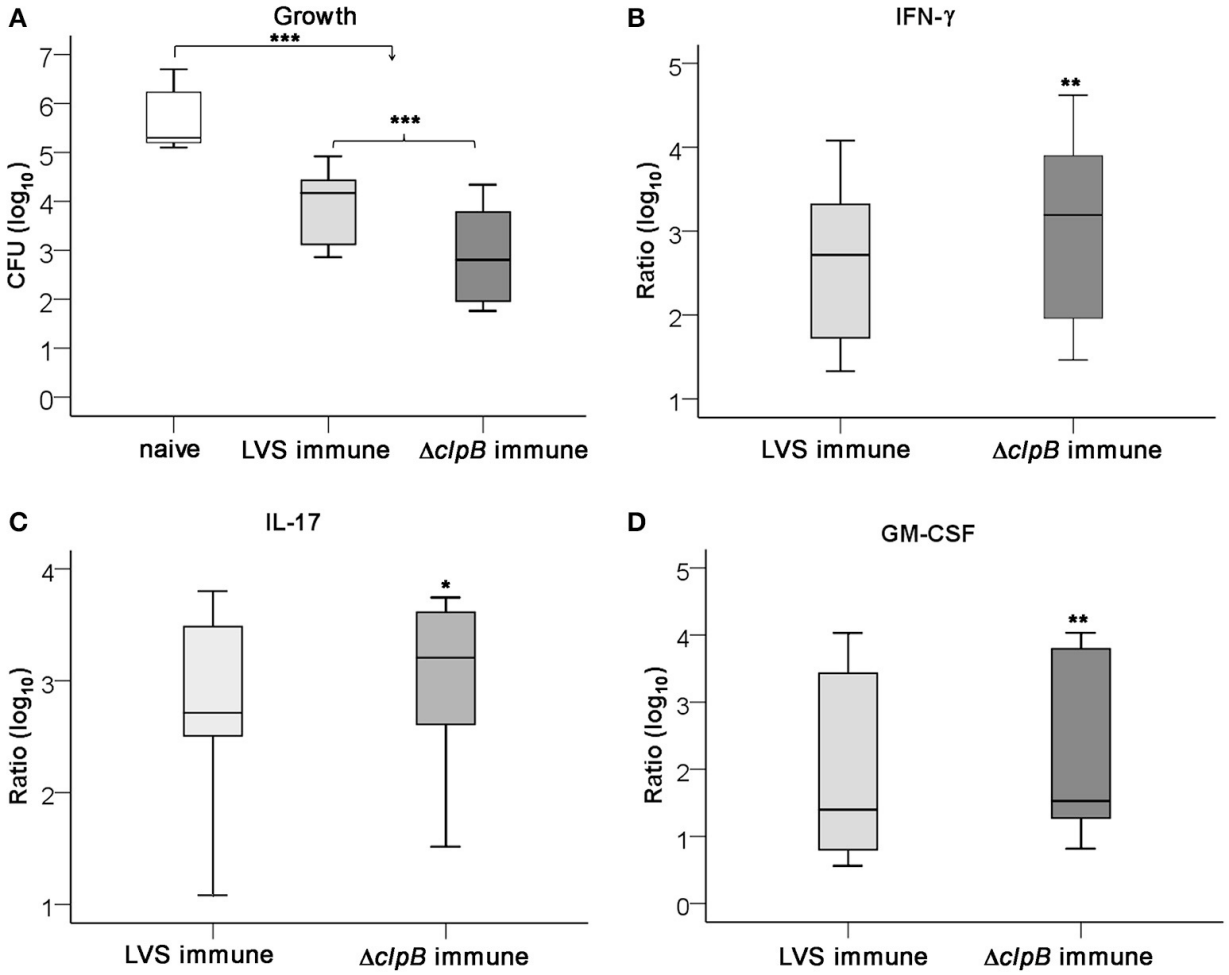
**Growth of ***F. tularensis*** SCHU S4 and cytokine accumulation in the co-culture assay**. Growth of SCHU S4 in cultures with naïve, LVS-immune or Δ*clp*B-immune splenocytes was determined after 72 h **(A)** and the accumulated levels of IFN-γ **(B)**, IL-17 **(C)**, and GM-CSF **(D)** in these cultures. Results shown represent data from nine experiments. Growth was calculated as the CFU (log_10_) of cultures at 72 h subtracted with the CFU (log_10_) of cultures at 0 h, 2.3 [2.1, 3.0]. In graphs **(B–D)**, the values are expressed as log_10_ fold-regulation of the indicated cytokine in relation to the concentration in cultures with naïve splenocytes. ^*^ *P* < 0.05, ^**^ *P* < 0.01, ^***^ *P* < 0.001 according to Wilcoxon's signed rank test. In graphs **(B–D)**, the significances indicate the pair-wise comparisons between LVS- and Δ*clp*B-immune data for each cytokine and in graph **(A)** for the indicated pair-wise comparisons.

In agreement with our present findings, the splenocyte-BMDM co-culture assay has previously been demonstrated to mediate efficient control of LVS infection, but few studies have analyzed the requirements for the control of virulent *F. tularensis* strains (Mahawar et al., 2013; Griffin et al., 2015). Therefore, the same protocol as previously described for the LVS infection was used, but the highly virulent strain, SCHU S4, was instead used to infect the cultures. As was demonstrated for the LVS infection, Δ*clp*B-immune splenocytes conferred superior control of SCHU S4 compared to LVS-immune splenocytes (*P* < 0.001; Figure 4A). In fact, the LVS-immune cells did not restrict the growth of SCHU S4 to the same extent as of LVS, median growth was 4.2 [3.1, 4.4] vs. 3.2 [2.4, 3.8], respectively (*P* < 0.001; Figures 2A, 4A). In contrast, Δ*clp*B-immune Balb/c-derived splenocytes conferred as efficient growth inhibition of SCHU S4 as of LVS, median growth was 2.8 [1.9, 3.8] and 2.4 [1.7, 3.4] log_10_ CFU, respectively (*P* > 0.05; Figures 2A, 4A). The cytokine profile of SCHU S4-infected cultures resembled that of LVS-infected cultures, but many of the cytokines were somewhat higher expressed in the cultures with SCHU S4 (Table S1). Thus, SCHU S4-infected cultures with Δ*clp*B-immune splenocytes contained higher levels of IFN-γ, GM-CSF, and IL-17 than the cultures with LVS-immune splenocytes (*P* < 0.01 for IFN-γ and GM-CSF and *P* < 0.05 for IL-17: Figures 4B–D).

In summary, LVS-immune cells did not control SCHU S4 as well as it controlled LVS, whereas Δ*clp*B-immune cells showed superior control and controlled both strains to a similar extent. Δ*clp*B-immune cells induced higher levels of IFN-γ, IL-17, and GM-CSF than did LVS-immune cells.

### Nitric oxide production in splenocyte-BMDM co-cultures

**Figure 5:**
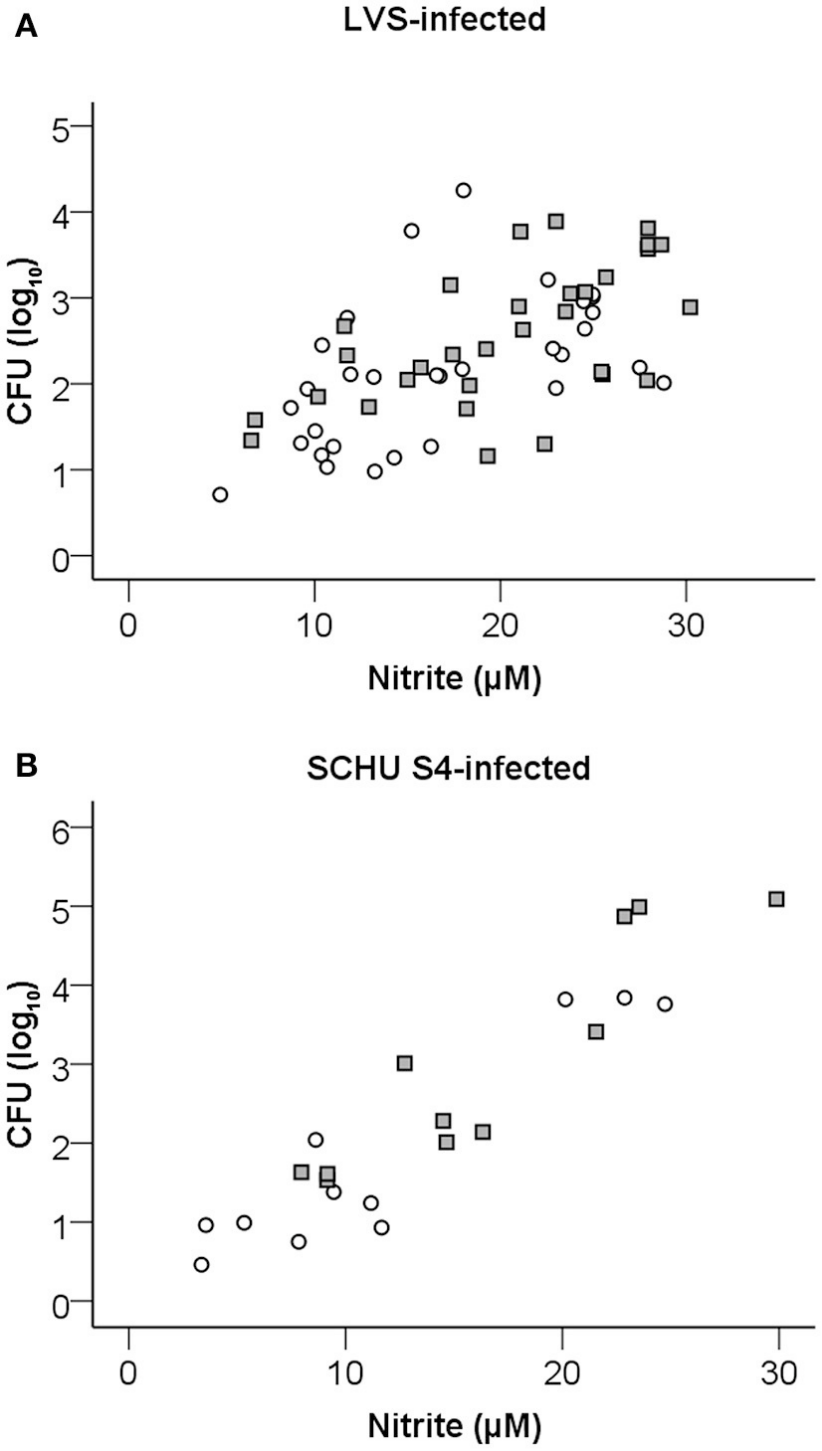
**Correlation of nitrite accumulation in cultures and growth inhibition of LVS (A)** or SCHU S4 **(B)** in the co-culture assay with LVS- (circles) or Δ*clpB*- (squares) immune splenocytes after 72 h of incubation. Results shown represent data from 13 experiments for LVS-infected cultures and five experiments for SCHU S4-infected cultures.

**Figure 6:**
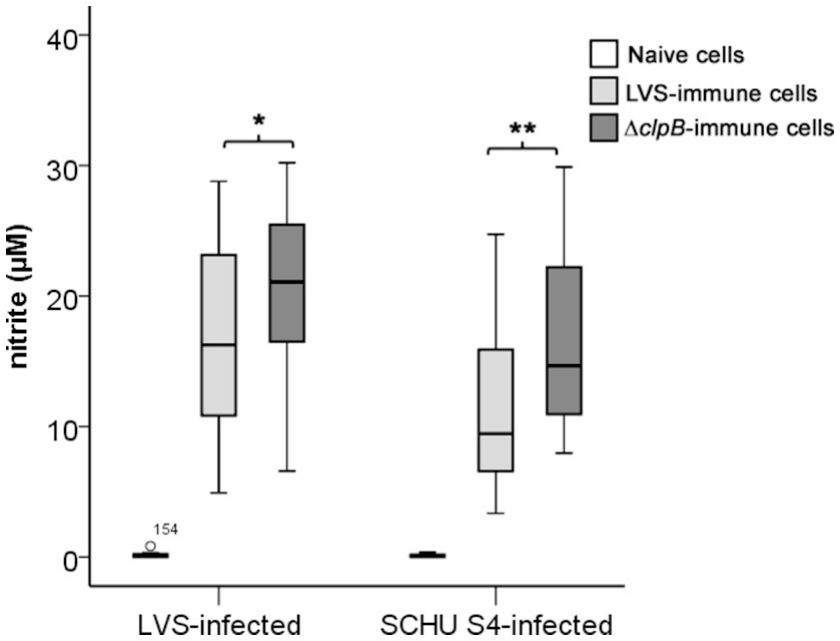
**Nitrite levels accumulated after 72 h in cultures with LVS- or SCHU S4-infected BMDM co-cultured with naïve (white boxes), LVS-immune (light gray boxes) or Δ***clp***B-immune (dark gray boxes) splenocytes**. Results shown represent data from 13 experiments for LVS-infected cultures and five experiments for SCHU S4-infected cultures. ^*^ *P* < 0.05, ^**^ *P* < 0.01 according to Wilcoxon's signed rank test for the indicated pair-wise comparisons.

Nitrite was measured in the supernatants of splenocyte-BMDM co-cultures infected with LVS and it was observed that the levels strongly correlated to growth inhibition (Spearman's rank correlation coefficient [rho] = 0.679, *P* < 0.01; Figure 5A). Nitrite levels were also highly correlated (*P* < 0.01) to levels of IFN-γ, GM-CSF, IL-6, IL-10, IL-12(p40), and RANTES (Table 1). Δ*clp*B-immune Balb/c-derived splenocytes stimulated higher nitrite production than did LVS-immune splenocytes, medians were 21 μM [16, 26] vs. 16 μM [10, 23], respectively (*P* < 0.05; Figure 6). Also when SCHU S4 was the infecting agent, nitrite levels strongly correlated to growth inhibition (rho = 0.745, *P* < 0.001; Figure 5B) and, again, cultures with Δ*clp*B-immune splenocytes displayed significantly higher levels of nitrite than did cultures with LVS-immune splenocytes, medians were 15 [9, 23] and 9 [5, 20] μM, respectively (*P* < 0.01; Figure 6).

In summary, these results demonstrate that Δ*clp*B-immune splenocytes showed superior capacity compared to LVS-immune splenocytes to stimulate nitrite production in the splenocyte-BMDM co-cultures and identify nitrite as a correlate of growth inhibition of both LVS and the highly virulent strain SCHU S4.

### Nitric oxide-dependent growth inhibition

**Figure 7:**
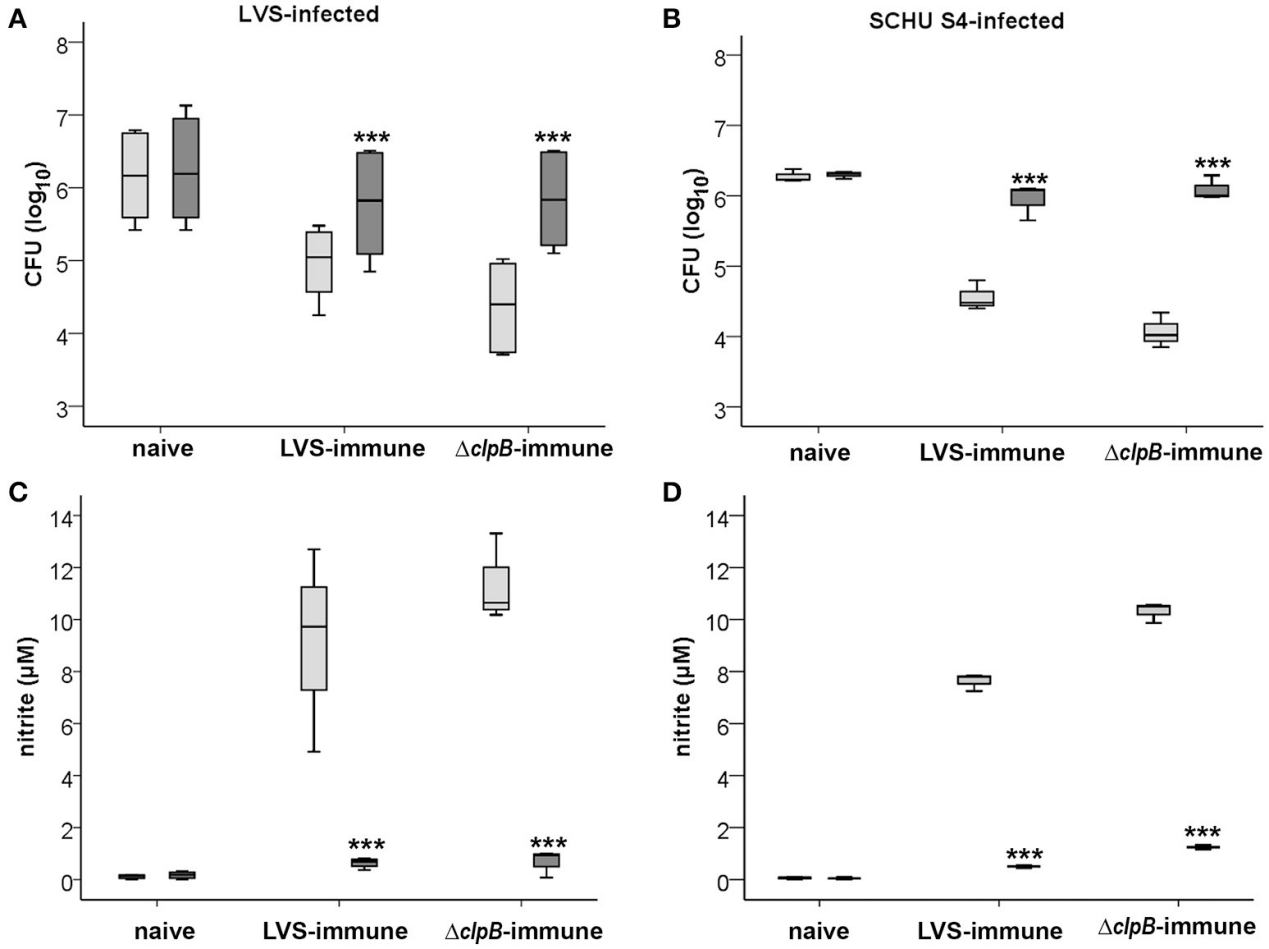
**Growth of ***F. tularensis*** (A,B)** and nitrite accumulation **(C,D)** in non-treated (light gray boxes) or NMMLA-treated (dark gray boxes) cultures. The data shown is from three experiments. ^***^ *P* < 0.001 according to Wilcoxon's signed rank test for the pair-wise comparisons between non-treated or NMMLA-treated cultures of each group.

Since nitrite levels correlated to the growth inhibition of *F. tularensis* effectuated by the immune splenocytes, it was further investigated if there was a direct correlation between the growth inhibition of *F. tularensis* and NO by use of the competitive inhibitor of iNOS, NMMLA. This treatment significantly reduced the growth inhibition of LVS or SCHU S4 conferred by the immune Balb/c-derived splenocytes (Figures 7A,B) and also reduced the nitrite production to about 1 μM compared to levels of at least 8.0 μM in its absence (Figures 7C,D). Levels in cultures with naïve splenocytes were consistently below 1 μM.

The role of NO was further investigated by use of BMDM derived from iNOS^−/−^ C57BL/6 mice. It should be noted that also these mice, like Balb/c mice, generate robust immunity after vaccination, since a majority survived an intradermal challenge of 1900 CFU and all of 5 mice survived 190 CFU after intradermal immunization with Δ*clp*B (Table 2), whereas the lethal dose for naïve mice was one CFU. In contrast, all of LVS-immunized mice succumbed to a challenge of 19 CFU within 22 days and maximal survival was even shorter when the challenge dose was 190 or 1900 CFU (Table 2). Thus, as for Balb/c mice (Conlan et al., 2010), immunization with Δ*clp*B confers superior protection in C57BL/6 mice.

**Table 2:** **Protective effect of LVS or Δ***clp***B immunization of C57/BL6 mice against intradermal challenge with SCHU S4**.

**Immunization^a^**	**Challenge dose^b^**	**Time of survival (days)**
LVS	19 CFU	7, 8, 9, 11, 22
Δ*clp*B	19 CFU	24, >33, >33, >33, >33
LVS	190 CFU	7, 8, 12, 12
Δ*clp*B	190 CFU	>33, >33, >33, >33, >33
LVS	1900 CFU	7, 8, 8, 9, 17
Δ*clp*B	1900 CFU	17, 22, 33, 33, 33
None	19 CFU	6, 6, 6, 6, 6

a Mice were intradermally immunized with 100,000 CFU of the LVS or the ΔclpB strain.

b Mice were 6 weeks after immunization challenged intradermally with the indicated dose of SCHU S4.

**Figure 8:**
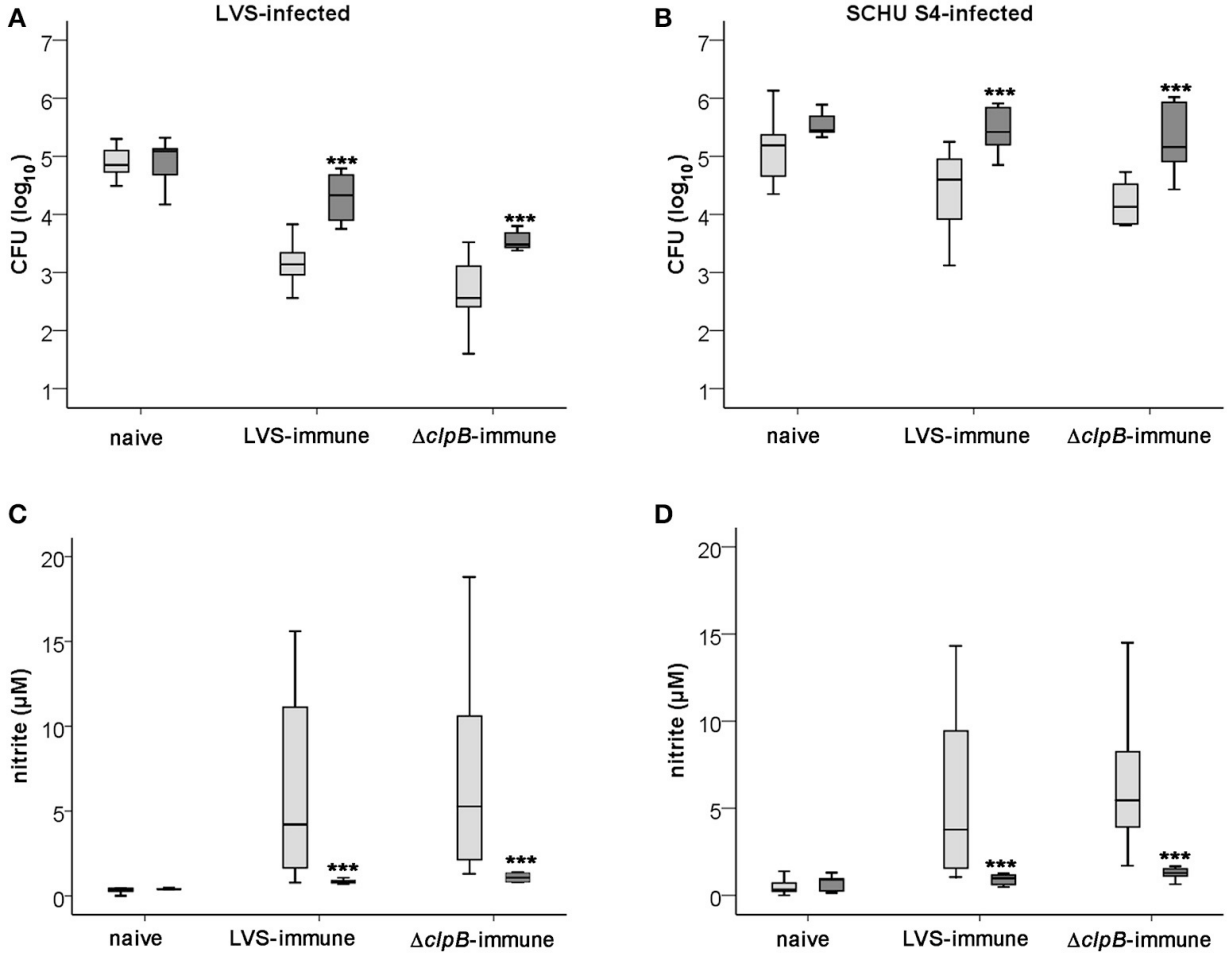
**Growth of ***F. tularensis*** (A,B)** and nitrite accumulation **(C,D)** in cultures with BMDM derived from C57BL/6 (light gray boxes) or iNOS^−/−^ (dark gray boxes) mice cocultured with naïve, LVS-immune or Δ*clp*B-immune splenocytes after 72 h. **(A)** and **(C)** represent LVS-infected cultures and **(B)** and **(D)** SCHU S4-infected cultures. Results shown in graphs A and B represent data from 6 experiments for iNOS^−/−^ BMDM and 8 experiments for C57BL/6 BMDM and in graphs C and D three experiments for iNOS^−/−^ BMDM and five experiments for C57BL/6 BMDM. ^***^ *P* < 0.001 according to Wilcoxon's signed rank test for the pair-wise comparisons between co-cultures with cells derived from C57BL/6 or iNOS^−/−^ mice of each group.

Similar to the splenocytes derived from Balb/cJ mice, C57BL/6-derived Δ*clp*B-immune splenocytes controlled growth of LVS and SCHU S4 better than did LVS-immune splenocytes, *P* < 0.05 and *P* < 0.01, respectively (Figures 8A,B), and induced higher nitrite production in the cultures, *P* < 0.01 and *P* < 0.05 for LVS- and SCHU S4-infected cultures, respectively (Figures 8C,D). Also in cultures with C57BL/6 cells, as in cultures with Balb/cJ cells, the nitrite levels were highly correlated to the growth inhibition of both LVS and SCHU S4 (rho = 0.745 and 0.771, respectively, *P* < 0.01). Further corroborating the role of NO, the growth inhibition elicited by the immune splenocytes was significantly lower (*P* < 0.001) in cultures with iNOS^−/−^ BMDM compared to C57BL/6 BMDM, whether or not the cultures were infected with LVS or SCHU S4 (Figures 8A,B). However, even in the absence of iNOS-derived NO, LVS- and Δ*clp*B-immune splenocytes still reduced the growth of LVS, median net growth were 4.3 [3.9, 4.7] and 3.5 [3.4, 3.7] log_10_ CFU, respectively, vs. 5.1 [4.7, 5.1] for naïve splenocytes; Δ*clp*B-immune splenocytes being significantly superior to LVS-immune splenocytes (*P* < 0.001; Figure 8A). In contrast, growth inhibition of SCHU S4 in cultures with iNOS^−/−^ BMDM was very minor or non-existent for both LVS- and Δ*clp*B-immune splenocytes (Figure 8B).

Collectively, the data obtained with C57BL/6-derived cells confirmed the results based on the Balb/c-derived cells, i.e., Δ*clp*B-immune splenocytes showed superior capacity compared to LVS-immune splenocytes to control the *F. tularensis* infection and the Δ*clp*B-immune splenocytes induced higher levels of nitrite production, which strongly correlated to growth inhibition. In addition, iNOS was found to be critically required for the growth inhibition of SCHU S4 and contributed significantly to the control of LVS.

## Discussion

It is well-established that protection against many intracellular pathogens is critically dependent on cell-mediated immunity, however, there are no methods validated to identify correlates of protection to these pathogens. In addition, for diseases like tularemia, which is infrequent in most parts of the world, the power of human clinical trials is unlikely to be sufficient to provide unambiguous data regarding the efficacy of vaccine candidates. To this end, the Animal Rule is an option for licensing of future tularemia vaccines and this option is most likely applicable to biodefense agents and sporadically occurring diseases, both of which are relevant to tularemia. Critically required in this regard is the identification of correlates of protection, i.e., a measurement of a biologically relevant function for and correlated to the degree of protection conferred in the animal model. For tularemia and other diseases caused by intracellular pathogens, e.g., tuberculosis, much focus has been on the role of IFN-γ as a correlate of protection. Although important for the protection *in vitro* to *Mycobacterium tuberculosis*, levels of the cytokine have not shown good correlation to protection in various clinical and experimental models (Goldsack and Kirman, 2007; Mittrücker et al., 2007; Jeevan et al., 2009). With regard to tularemia, IFN-γ appears to be necessary, although not sufficient for protection (Elkins et al., 2007; Conlan, 2011). Thus, there is a need to develop models to better define the correlates of protection against tularemia.

The use of the splenocyte-BMDM co-culture model demonstrates distinct advantages compared to commonly used animal models for tularemia, since the number of animals required will be far less and the mice that are immunized as a source of splenocytes receive a sublethal dose that results in few or no objective symptoms. Thus, the method leads to both distinct reduction of the number of animals required, as well as refinement, since the distress caused by the sublethal infection will be minimal. In addition, the use of the co-culture model allows for direct comparisons of correlates with those identified in human models. In fact, a human co-culture model, based on the use of adherent and non-adherent peripheral blood mononuclear leukocytes from LVS-vaccinated individuals or former tularemia patients, shows promise in this regard (Eneslätt et al, unpublished). Since it is likely that human challenge studies never will be performed in the future, the use of a human co-culture model may be the only realistic way to identify correlates of immunity and protection. A limitation of co-culture models is there will be a selection of cell types included and the model may therefore not be representative for certain organ-specific immune responses *in vivo*. It should be noted that there are examples when organ-specific *F. tularensis* co-culture models have been utilized to overcome this limitation (De Pascalis et al., 2014).

Although previous studies based on splenocyte-BMDM co-culture models have revealed much about the vaccine-mediated protection; both the prerequisites for protection against LVS as well as putative correlates of protection, there have been very few studies with virulent *F. tularensis* strains based on a co-culture system (Mahawar et al., 2013; Griffin et al., 2015).

The present study used the LVS strain as benchmark for *F. tularensis* vaccine efficacy. Vaccination with LVS effectively prevents laboratory-acquired infection, but studies on volunteers have revealed that it affords only marginal protection against aerosol infection (Saslaw et al., 1961a,b; Burke, 1977). It was hypothesized that it would be possible to generate a superior *F. tularensis* vaccine and we and others have generated a number of targeted mutants of SCHU S4 and their efficacy has been evaluated using *in vivo* models with virulent *F. tularensis* strains (Kadzhaev et al., 2009; Conlan et al., 2010; Shen et al., 2010; Ryden et al., 2012; Santiago et al., 2015). In particular one mutant, Δ*clpB*, was found to confer superior efficacy to LVS, as demonstrated by survival after infection with SCHU S4 (Conlan et al., 2010). In agreement with the findings in both Balb/c and C57BL/6 mice, our findings using the splenocyte-BMDM co-culture method demonstrate that the Δ*clpB*-immune splenocytes were superior to LVS-immune splenocytes in several aspects, most notably that the Δ*clp*B-immune splenocytes conferred superior control of both LVS and SCHU S4 infection. In addition, LVS-immune splenocytes conferred significantly less control of SCHU S4 than LVS. Likewise, a previous study demonstrated that LVS-immune splenocytes controlled SCHU S4 infection only when pre-stimulated *in vitro* (Griffin et al., 2015).

Although previous studies based on various splenocyte-BMDM co-culture models have revealed much about the vaccine-mediated protection; both the prerequisites for protection against LVS as well as putative correlates of protection, there have been very few studies with virulent *F. tularensis* strains based on a co-culture system (Mahawar et al., 2013; Griffin et al., 2015) and, therefore, the present study provides important information regarding the protective mechanisms operative against the highly virulent SCHU S4 strain. In agreement with the previous studies (Mahawar et al., 2013; Griffin et al., 2015), our findings demonstrate a strong correlation between the ability of the vaccine strains to confer protection to virulent strains *in vivo* and their capability to efficiently prime the protective efficacy of the immune cells as measured by the splenocyte-BMDM co-culture model. In addition, our study reveals that the quality of the immune responses elicited by LVS and Δ*clp*B are similar, although the response elicited by the Δ*clp*B vaccine is quantitatively more robust.

Multi-parameter flow cytometry has been used rather extensively to characterize memory T cells and the technique has enabled detailed descriptions of their phenotypic characteristics and functional abilities. In various experimental models, it has been argued that specific polyfunctional T cells demonstrate good correlation with host protection. While promising in the context of mouse models of *Leishmania* and certain tuberculosis vaccines (Darrah et al., 2007; Derrick et al., 2011), polyfunctional T cells have in other cases failed to demonstrate correlation to protection (Connor et al., 2010; Kagina et al., 2010; Harari et al., 2011). Human responses to killed *F. tularensis* antigens from the LVS or SCHU S4 strains have been characterized using multi-parameter flow cytometry and it was found that IFN-γ and MIP-1β strongly discriminated between immune and naïve individuals (Eneslätt et al., 2012). Also in the present study, we found evidence that the immune cell populations contained polyfunctional T cells, expressing combinations of IFN-γ, TNF-α, and IL-17. Interestingly, the relative frequencies of all variants of polyfunctional CD4^+^ and CD8^+^ T cells expressing IFN-γ was consistently higher among Δ*clp*B- than LVS-immune splenocytes.

Also in other important aspects, there were quantitative differences between the immune responses triggered by the Δ*clp*B- *vs*. the LVS-immune splenocytes, since the Δ*clp*B-immune splenocytes demonstrated stronger proliferative responses and higher production of NO, IFN-γ, GM-CSF, and IL-17, all of which correlated to growth inhibition. Altogether, all of these properties of the Δ*clp*B-immune splenocytes, together with their superior ability to confer growth inhibition of SCHU S4, help to explain the efficaciousness of Δ*clp*B. An interesting finding was that IFN-γ was the most strongly induced cytokine after addition of immune splenocytes, whether or not they were derived from Δ*clp*B- or LVS-immunized mice. Considering the critical role of IFN-γ for many aspects of immunity to *F. tularensis*, the finding shows that the splenocyte-BMDM co-culture system used herein mirrors relevant aspects of other models of tularemia.

The effect of adding splenocytes derived from either C57BL/6 or Balb/cJ was also analyzed. In both instances, Δ*clp*B-immune splenocytes showed superior capacity compared to LVS-immune splenocytes to control the *F. tularensis* infection and also to induce production of higher levels of NO. This demonstrates that the distinct differences between Δ*clp*B-immune and LVS-immune splenocytes were not affected by the genetic background of the splenocytes.

It has been shown that control of LVS in a macrophage-BMDM co-culture assay correlated with levels of nitrite (Bosio and Elkins, 2001; Elkins et al., 2009). Our results using the co-culture method provided strong evidence for the critical role of NO for control also of the SCHU S4 infection as supported both by experiments using an inhibitor of iNOS, as well as iNOS-deficient BMDM. Although this may appear somewhat paradoxical, considering that NO plays a minor or insignificant role *in vitro* (Lindgren et al., 2005; Edwards et al., 2010), it is likely that NO production in the rather complex splenocyte-BMDM co-culture model used herein confers additional effects compared to the model based on monocytic cells only. In further support of the important role of NO, it exerts a critical function *in vivo*, since iNOS-deficient mice succumb even to the lowest inocula of an LVS infection (Lindgren et al., 2004).

The enhanced NO production in Δ*clp*B cultures was likely dependent on the higher secretion of IFN-γ and IL-17, and the higher frequency of polyfunctional T cells, expressing combinations of IFN-, TNF-α, and IL-17, since these cytokines regulate iNOS. Regulation of iNOS is in many aspects distinct among species, but the critical roles of IFN-γ, TNF-α, IL-1β, and IL-17 to induce its expression appears to be conserved regardless of species (Pautz et al., 2010; Mühl et al., 2011). Many studies have demonstrated a critical role of NO in various murine infectious models, but there is also significant evidence supporting its role for control of human infectious diseases. One notable example is tuberculosis, the etiological agent of which is a facultative intracellular bacterium, as for tularemia. It has been observed that human *M. tuberculosis*-infected monocytes produce NO and this results in a bacteriostatic, and sometimes even bactericidal effect (Landes et al., 2015). Further corroborating an important role of NO, it has been observed that iNOS expression is increased in human granulomas (Mattila et al., 2013). Malaria is another important human disease where there is evidence for a critical role of NO, since endogenous production of asymmetrical dimethylarginine, an iNOS inhibitor, strongly correlated to mortality (Yeo et al., 2010). Collectively, such findings suggest that NO could have an important role in other human diseases, such as tularemia, and, therefore, that the findings regarding the superior protection conferred by Δ*clp*B in the mouse model also will have relevance for the human situation.

Overall, our results provide new information as to why Δ*clp*B is superior to LVS as a vaccine for protection against highly virulent *F. tularensis*. The information is critical for the understanding of protective mechanisms and thereby for important for licensing of future vaccines against this potent pathogen.

## Author contributions

Conceived and designed the experiments: IG, HL, KE, AS, Performed the experiments: IG, HL, KE, AM, WC, Analyzed the data: IG; HL, KE, WC, AS, Contributed reagents/materials/analysis tools: AM, TH. Wrote the paper: IG, HL, TH, AS.

## Funding

Grant support was obtained from the Swedish Medical Research Council (K2012-3469 and K2013-8621), Västerbottens läns landsting (Spjutspetsmedel, VLL-582571, Centrala ALF medel, VLL-463691), the Medical Faculty, Umeå University, Umeå, Sweden, and from the ERC (ERC starting grant 311542 to TH). The work was performed in part at the Umeå Centre for Microbial Research (UCMR).

### Conflict of interest statement

The authors declare that the research was conducted in the absence of any commercial or financial relationships that could be construed as a potential conflict of interest. The reviewer TEP and handling Editor declared their shared affiliation and the handling Editor states that the process nevertheless met the standards of a fair and objective review.
