# Supplementary material for: An In Vitro Co-culture Mouse Model Demonstrates Efficient Vaccine-Mediated Control of Francisella tularensis SCHU S4 and Identifies Nitric Oxide as a Predictor of Efficacy
Source: Front Cell Infect Microbiol. 2016 Nov 25;6:152. doi: 10.3389/fcimb.2016.00152 (PMC5122580; doi:10.3389/fcimb.2016.00152)
Supplement: Supplementary file 1 [file Table1.DOCX]

**SUPPLEMENTARY DATA**

Table S1. Fold cytokine accumulation in the cultures with LVS- or ∆*clp*B-immune cells compared to cultures with naïve cells after 72 h.

| Cytokine | LVS-infected | | SCHU S4-infected | |
| --- | --- | --- | --- | --- |
|  | LVS immune | *∆clp*B immune | LVS immune | *∆clp*B immune |
| IL-1α | 24 ± 12 | 19 ± 6 | 11 ± 3 | 14 ± 6 |
| IL-1β | 4 ± 1 | 4 ± 2 | 3 ± 1 | 3 ± 2 |
| IL-2 | 130 ± 55 | 124 ± 49 | 124 ± 61 | 176 ± 64 |
| IL-3 | 5 ± 3 | 5 ± 2 | 7 ± 4 | 7 ± 3 |
| IL-4 | 3 ± 1 | 2 ± 1 | 5 ± 2 | 3 ±1 |
| IL-5 | 4 ± 2 | 4 ± 1 | 26 ± 20 | 17 ± 10 |
| IL-6 | 98 ± 17 | 128 ± 27 | 38 ± 15 | 63 ± 30 |
| IL-9 | 2 ± 2 | 1 ± 1 | 2 ± 1 | 2 ± 1 |
| IL-10 | 2 ± 1 | 2 ± 1 | 2 ± 1 | 2 ± 1 |
| IL-12(p40) | 26 ± 10 | 29 ± 12 | 8 ± 4 | 11 ± 6 |
| IL-12(p70) | 5 ± 3 | 5 ± 3 | 5 ± 2 | 8 ± 4 |
| IL-13 | 2 ± 1 | 2 ± 0 | 2 ± 1 | 2 ± 1 |
| IL-17 | 31 ± 6 | 51 ± 12 | 213 ± 111 | 368 ± 151 |
| Eotaxin | 4 ± 3 | 3 ± 2 | 3 ± 1 | 7 ± 4 |
| G-CSF | 17 ± 14 | 9 ± 5 | 5 ± 0 | 4 ± 1 |
| GM-CSF | 10 ± 3 | 14 ± 3 | 1869 ± 990 | 3065 ± 1550 |
| IFN-γ | 730 ± 178 | 1360 ± 408 | 2197 ± 1402 | 7107 ± 4989 |
| KC | 5 ± 4 | 3 ± 1 | 2 ± 0 | 1 ± 0 |
| MCP-1 | 19 ± 14 | 9 ± 5 | 4 ± 1 | 3 ± 1 |
| MIP-1α | 0.2 ± 0.2 | 0.4 ± 0.3 | 3 ± 2 | 1 ± 1 |
| MIP-1β | 3 ± 1 | 3 ± 1 | 2 ± 1 | 3 ± 2 |
| RANTES | 221 ± 107 | 141 ± 54 | 197 ± 175 | 394 ± 158 |
| TNF-α | 1 ± 0 | 2 ± 1 | 3 ± 1 | 4 ± 1 |
